# The Hippo Pathway in Prostate Cancer

**DOI:** 10.3390/cells8040370

**Published:** 2019-04-23

**Authors:** Omar Salem, Carsten G. Hansen

**Affiliations:** 1 Queen’s Medical Research Institute, University of Edinburgh Centre for Inflammation Research, Edinburgh bioQuarter, 47 Little France Crescent, Edinburgh EH16 4TJ, UK; omar.m.salem@ed.ac.uk; 2 Institute for Regeneration and Repair, University of Edinburgh, Edinburgh bioQuarter, 5 Little France Drive, Edinburgh EH16 4UU, UK

**Keywords:** hippo pathway, YAP/TAZ, prostate cancer, castration resistance, signal cross-talk, feedback loops

## Abstract

Despite recent efforts, prostate cancer (PCa) remains one of the most common cancers in men. Currently, there is no effective treatment for castration-resistant prostate cancer (CRPC). There is, therefore, an urgent need to identify new therapeutic targets. The Hippo pathway and its downstream effectors—the transcriptional co-activators, Yes-associated protein (YAP) and its paralog, transcriptional co-activator with PDZ-binding motif (TAZ)—are foremost regulators of stem cells and cancer biology. Defective Hippo pathway signaling and YAP/TAZ hyperactivation are common across various cancers. Here, we draw on insights learned from other types of cancers and review the latest advances linking the Hippo pathway and YAP/TAZ to PCa onset and progression. We examine the regulatory interaction between Hippo-YAP/TAZ and the androgen receptor (AR), as main regulators of PCa development, and how uncontrolled expression of YAP/TAZ drives castration resistance by inducing cellular stemness. Finally, we survey the potential therapeutic targeting of the Hippo pathway and YAP/TAZ to overcome PCa.

## 1. Introduction

**Figure 1:**
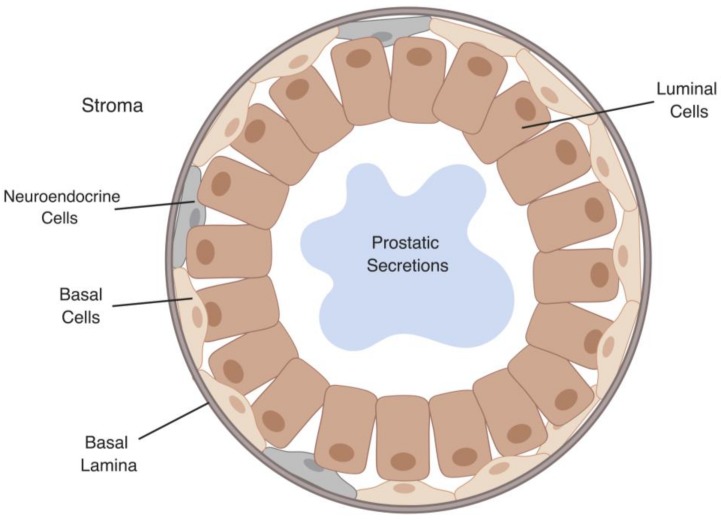
Representation of different cell types in the prostate gland.

Prostate cancer (PCa) is worldwide one of the most prevalent cancers in men, with over one million new cases reported annually [1,2]. Initially, premalignant prostatic intraepithelial neoplasia (PIN) lesions form, which develop into advanced localized PCa followed by metastasis [3,4]. The prostate gland consists of luminal, basal, and neuroendocrine cells embedded in fibromuscular stroma (Figure 1) [4,5]. The most commonly reported PCa is acinar adenocarcinoma, which is androgen receptor (AR)-positive and arises from the prostate gland secretory luminal cell lineage [4,5]. A smaller subset of PCa develops from the neuroendocrine cell lineage [4,5]. Neuroendocrine tumors are classified as small-cell carcinoma and are more prevalent following recurrence [4,5] (Figure 1).

Early stages of prostate cancer are managed by surveillance, as well as classical approaches such as radiation therapy and surgery [6,7]. However, the first line of treatment of locally advanced or metastatic prostate cancer is androgen deprivation therapy (ADT) [8,9,10]. Although ADT is effective initially, patients develop castration-resistant prostate cancer (CRPC) within 1–3 years. CRPC is defined as PCa that progressed despite castrate serum testosterone levels (<50 ng/dL) [11,12].

Clinical management of CRPC is challenging, which is partly due to the molecular variation between patients [13]. Several mechanisms activate AR in CRPC patients [12,14]. These include AR mutations and amplification, which leads to AR hypersensitivity or promiscuity, causing the activation of AR in response to low androgen levels and non-androgenic steroids [15,16]. PCa expressing some AR splice variants also overcomes ADT. These alternative AR variants are constitutively active due to the loss of the C-terminal part of the AR ligand-binding domain [14,17]. Additionally, CRPC patients have relatively higher androgen levels compared to healthy males [18], which is due to intratumoral steroidogenesis, as well as altered adrenal steroid production [18,19]. Notably, ligand-independent activation of AR also plays prominent roles in CRPC [20]. Despite recent efforts to optimize current ADT strategies, CRPC remains a global burden. Advanced PCa is characterized by poor prognosis and high mortality rate, causing approximately 350,000 global deaths annually [1,2]. There is, therefore, an urgent need to unravel the complex mechanism underlying PCa development, progression, and ADT resistance in order to identify new druggable targets.

The Hippo signaling pathway is a major player in stem cells and cancer biology [21,22]. The Hippo signaling cascade, identified through studies of tumor suppressors in the fruitfly, *Drosophila melanogaster* [23], is conserved across species, including humans [24]. It acts as a crucial regulator of cell growth and proliferation, organ development, cellular homeostasis, and regeneration [22,25]. The Hippo pathway is regulated by multiple signals such as, cell-density/polarity, mechanotransduction, nutrients, and via G-protein-coupled receptors [26,27,28,29]. Importantly, apparent kinase cascade independent regulation of Yes-associated protein (YAP)/ transcriptional coactivator with PDZ-binding motif (TAZ) also takes place [30,31,32] (Box 1). The upregulation of the Hippo pathway downstream effectors, YAP/TAZ, is central in a variety of solid tumors [21,25,29,33,34]. Prominently, the implications of elevated activity of YAP/TAZ in prostate cancer (PCa) are becoming apparent.

In this review article, we summarize the expanding evidence linking YAP and TAZ to PCa development, hormone inhibition resistance, and metastasis. Additionally, we highlight the role of the Hippo pathway in regulating prostate cancer stem cells and the importance of Hippo–YAP/TAZ as a potential therapeutic target for PCa, and we stress hitherto outstanding questions of how the dysregulated Hippo pathway drives PCa onset and development.

Box 1 **Yes-associated protein (YAP)/PDZ-binding motif (TAZ) Regulation by the Canonical Hippo Pathway.**

The Hippo pathway consists of an upstream serine-threonine kinase cascade. The chief kinases are MST1/2 (the mammalian Hippo homolog) and the MAP4K family of kinases, which phosphorylate and, in turn, activates large tumor suppressor (LATS1/2) [35,36,37,38,39,40,41,42,43,44,45]. When the Hippo kinases are “active”, LATS1/2 phosphorylate and thereby inhibit the transcriptional co-activator YAP [46] and its paralog TAZ [47], causing their cytoplasmic retention by protein 14-3-3, AMOT, or degradation [30,48,49,50,51]. In contrast, when the kinase module is “inactive”, dephosphorylation of YAP/TAZ occurs, which allows YAP/TAZ to translocate to the nucleus and regulate transcription. YAP/TAZ-mediated transcriptional regulation is predominantly via direct binding to the transcription factors TEAD1–TEAD4 [52,53,54]. As a consequence, the expression of multiple proliferative and antiapoptotic genes occurs, such as *connective tissue growth factor (CTGF)* and *cysteine-rich angiogenic factor (CYR61)* [52,53,54]. Additional kinases were also shown to directly phosphorylate and thereby regulate YAP/TAZ, such as SRC [55,56,57,58], Nuclear Dbf2-related 1/2 (NDR1/2) [59], c-Jun N-terminal kinase (JNK) [60,61], 5’ adenosine monophosphate-activated protein kinase (AMPK) [62,63,64], and Nemo-like kinase (NLK) [65,66]. Finally, kinase-independent regulation of YAP/TAZ is also taking place [30,31,32].

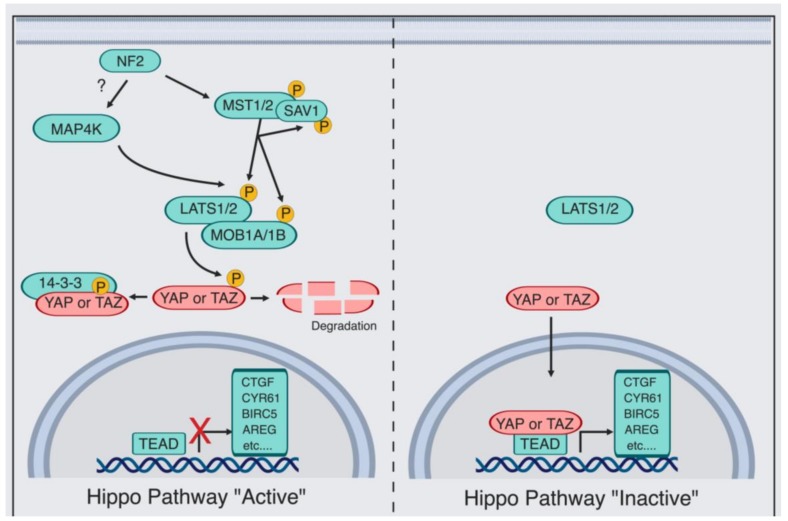

## 2. Hippo/YAP Key Players in Early Stages of Prostate Cancer

**Figure 2:**
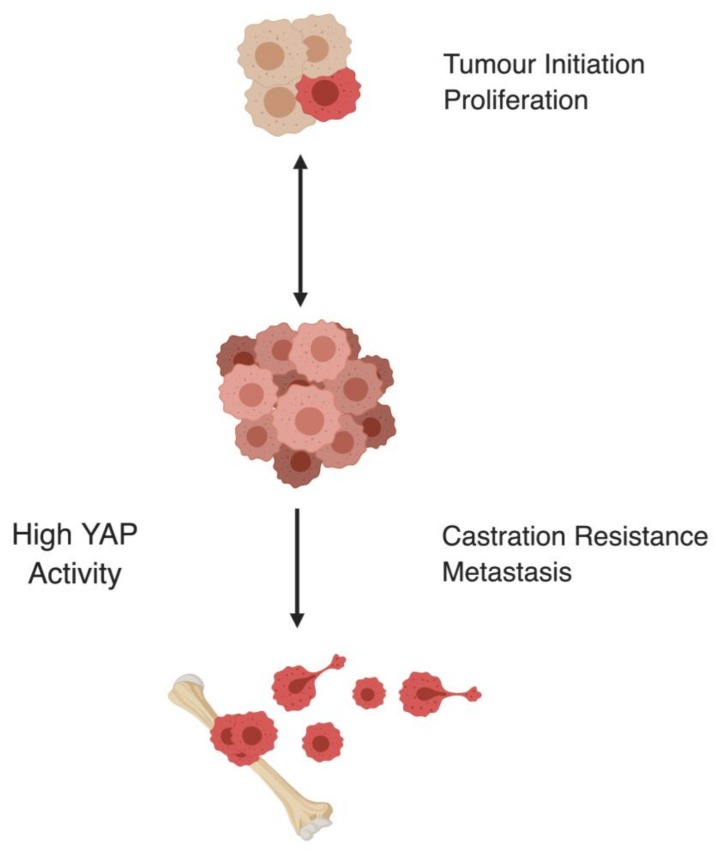
Schematic overview of YAP activity levels across different stages of prostate cancer (PCa). YAP regulates multiple stages of PCa [68,70,71].

Elevated YAP activity is observed in most solid tumors [34], and hyperactive YAP induces the formation of several carcinomas including liver, lung, breast, sarcoma, and pancreas [21,22,33,67]. YAP is also identified as a clinical marker for PCa progression [68] and regulator of CRPC [69]. YAP levels correlate with patients’ Gleason score, prostate-specific antigen (PSA) levels, and extraprostatic extensions [68,70] (Figure 2).

**Figure 3:**
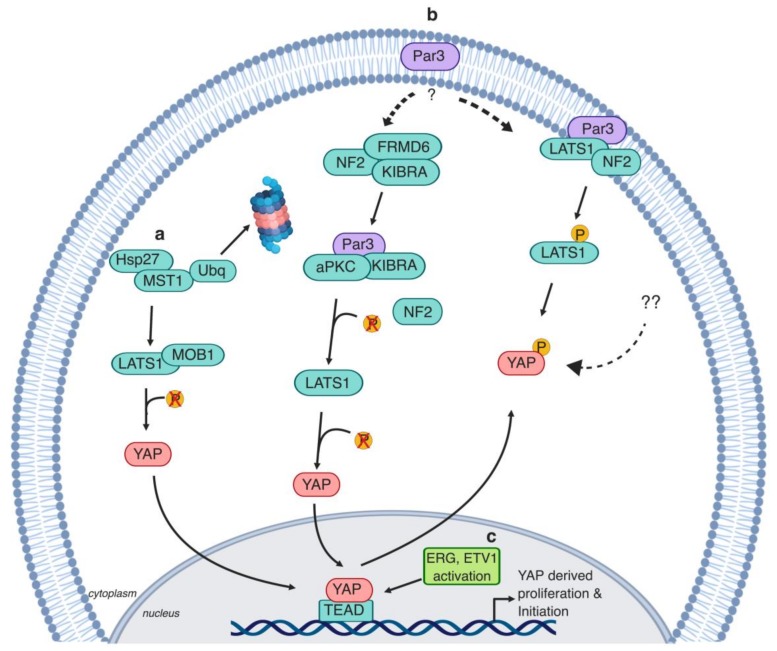
Mechanisms of YAP regulation in early stages of prostate cancer. **a.** Heat shock protein 27 (Hsp27) induces MST1 ubiquitin-mediated degradation, which in turn causes LATS1 and MOB1 dephosphorylation and thereby inactivation, consequently inducing YAP nuclear translocation [88]. **b.** Two different mechanisms were proposed by which polarity protein (Par3) regulates YAP; (1) Par3 inhibits YAP activity through inducing the recruitment of Neurofibromatosis type 2 (NF2/Merlin) and LATS1 to the membrane. As a result, LATS1 is activated, which induces YAP phosphorylation and cytoplasmic retention [83]. (2) Par3 induces YAP activation through the dissociation of kidney- and brain-expressed protein (KIBRA) from its canonical complex (KIBRA/NF2/ FERM domain-containing protein 6 (FRDM6)) and drives the recruitment of KIBRA to the Par3/aPKC/KIBRA complex. Thus, the interaction between KIBRA and LATS1 is disrupted, which induces LATS1 dephosphorylation and thereby YAP activation [84]. **c.** E26 transformation-specific (ETS) transcription factors trigger YAP induction. (1) ETS-regulated gene (ERG) activation drives YAP activation in old aged mice. ERG induces YAP and TEAD4 promoter activity and thereby triggers YAP target gene expression [75]. (2) ETS translocation variant 1 (ETV1) drives *YAP* activation by recruiting lysine specific demethylase (JMJD2A) to the *YAP* promoter [77].

Additionally, exogenous overexpression of YAP in normal prostate epithelial cells induces colony formation and increased migration in three-dimensional (3D) cultures [71]. How YAP becomes hyperactivated and drives PCa initiation and development is currently not clear, but several mechanisms were recently implicated (Figure 3).

### 2.1. E26 Transformation-Specific (ETS) Transcription Factors

ETS-regulated gene (ERG) is a transcription factor that belongs to the E26 transformation-specific (ETS) family and drives proliferation, apoptosis, and angiogenesis [72]. ERG overexpression in PCa results due to the fusion on chromosome 21q22 between the first exon of the androgen regulated gene *TMPRSS2* and the coding sequence of *ERG* [72,73]. This is a relatively frequent translocation, present in approximately 40–50% of PCa patients [74]. ERG overexpression results in the development of PCa tumors in aged mice [75]. Mechanistically, ERG induces *YAP* promoter activity in the hormone refractory PCa cell model (VCaP cells) [76], and ERG knockdown results in a decrease in YAP protein levels [75] (Figure 3). ERG both transactivates TEAD4 and directly binds to the *CTGF* promoter region, thereby inducing *CTGF* expression [75]. ETV1, an additional member of the ETS transcription factors, induces *YAP* expression in LNCaP cells by recruiting the lysine-specific demethylase (JMJD2A) to the *YAP* promoter [77] (Figure 3). ETV1-induced *YAP* expression in vivo causes PIN lesion formation, which, when combined with a single copy loss of phosphatase and tensin homolog (PTEN), progresses to malignant carcinoma [77]. PTEN is a negative regulator of the phosphoinositide 3-kinase/protein kinase B (PI3K/AKT) pathway, which controls proliferation and apoptosis [78]. PTEN deletions are identified in around 20% of primary PCa and 50% of advanced PCa [79].

### 2.2. Polarity Protein (Par3)

Epithelial cells are polarized cells with distinct functional apical and basolateral membrane domains [80]. Par3, among other polarity proteins, is a major regulator of epithelial cell structure and function [81,82]. Moreover, loss of Par3 is present in a variety of epithelial tumors [82]. Par3 loss leads to the formation of high-grade PIN lesions in vivo due to high YAP activity [82]. In this context, Par3 interrupts the NF2-derived recruitment of LATS1 to the plasma membrane [83]. Additionally, PIN lesions progress to PCa adenocarcinoma when combined with LATS1 loss in the Par3/LATS1 knockout (KO) murine model [83]. Contradictorily, Par3 sequesters the potent Hippo kinase cascade activator, kidney- and brain-expressed protein (KIBRA), and prevents it from forming a complex with NF2 [84]. As a result, KIBRA complexes with Par3 and atypical protein kinase C (aPKC). Knockdown of Par3 expression in PCa cells restores LATS1 and YAP phosphorylation levels, resulting in a lower migration rate in vitro and lower rate of metastasis in vivo [84]. The findings from both studies suggest that Par3 expression is lost during tumor initiation, but might be retained in advanced PCa triggering metastasis [83,84] (Figure 3). The regulation of Par3 in PCa is not fully understood and the interplay between the Hippo pathway and polarity proteins in PCa requires further investigation.

### 2.3. Heat Shock Proteins

Heat shock proteins (Hsps) are cellular stress modalities that regulate signaling and homeostasis [85]. Hsp expression is upregulated in response to chemotherapy and hormonal therapy [86]. The heat shock protein 27 (Hsp27) is elevated in a variety of tumors such as lung, breast, and cervical cancers [86]. Hsp27 is utilized by PCa tumor cells to resist apoptosis following androgen deprivation [87]. Hsp27 acts as a regulator of MST1 via promoting its ubiquitin-mediated degradation. As a result, LATS1 and MOB kinase activator 1 (MOB1) phosphorylation are reduced, causing YAP dephosphorylation and nuclear translocation [88] (Figure 3). However, it is worth noting that ablation of both MST1 and MST2 is needed both in vivo and in vitro to generally impair LATS1/2 activation [89,90,91], and, in some contexts, inhibition of the MAP4K family of kinases is also necessary to reduce overall LATS1/2 activity and thereby to increase YAP activity [37,42,45].

## 3. The Hippo Pathway Promotes Castration Resistance and Metastasis in Prostate Cancer

### 3.1. Androgen Receptor—Regulator of CRPC Progression

The androgen receptor (AR) is a transcription factor that belongs to the superfamily of steroid receptor hormones [92]. AR signaling is essential for prostate development and homeostasis [93]. During absence of androgens, inactive AR resides in the cytoplasm bound to heat shock proteins [92]. Upon binding of dihydrotestosterone (DHT), AR dissociates and translocates to the nucleus to induce gene expression [92]. AR-mediated gene expression occurs via multiple AR coactivators and AR-mediated recognition of androgen response elements (AREs) on the target gene promoter. AREs consist of two common inverted hexameric half-sites (5′–AGAACA–3′) separated by three base pairs [94]. In healthy tissue, tight homeostatic androgen signaling between stromal and epithelial cells regulate the prostate gland function [93,95]. Disrupted AR signaling is a key event in PCa initiation, progression, and development of castration resistance [5,96,97]. However, to date, the exact molecular mechanism via which CRPC develops is yet to be fully explored [93].

### 3.2. AR and YAP Colocalization

**Figure 4:**
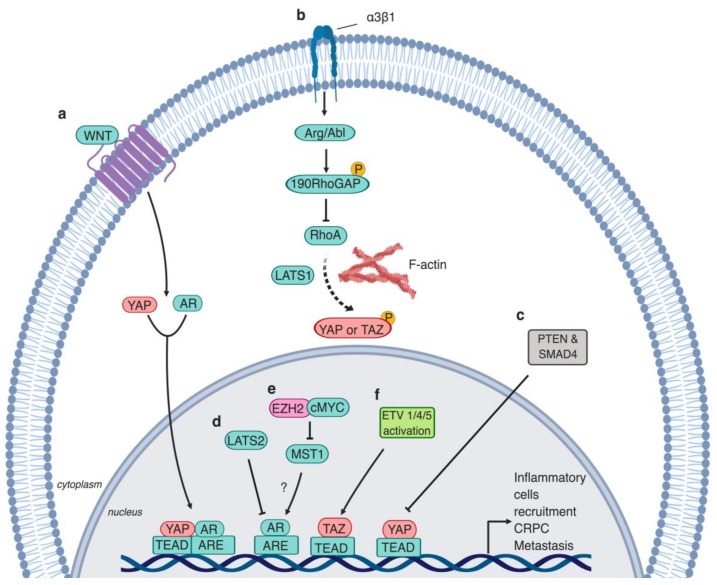
The Hippo pathway regulation of advanced prostate cancer. **a.** During androgen deprivation, Wingless (WNT) signaling drives the nuclear translocation of YAP and AR, resulting in YAP and AR target gene induction [143]. Additionally, YAP and AR colocalize in the nucleus and induce gene expression independently from WNT signaling or androgen availability [98]. **b.** α3β1 integrin stimulates the kinase activity of Arg/Abl, which phosphorylates 190RhoGAP, resulting in the inhibition of RhoA GTPases. Consequently, YAP/TAZ are phosphorylated via LATS1 activity and/or actin rearrangement and retained in the cytoplasm. α3β1 loss results in the disruption of this signaling cascade, inducing prostate cellular migration and metastasis [127]. **c.** PTEN and SMAD4 activity loss results in YAP hyperactivation. YAP signaling induces the recruitment of inflammatory cells [131]. **d.** LAST2 impedes AR receptor activity and restricts the binding of AR to the *prostate-specific antigen (PSA)* promoter [108]. **e**. EZH2 and c-MYC cooperate to induce methylation and silencing of *MST1*, which might induce AR activity [101]. **g.** ETV1/4/5 activate *TAZ*, triggering metastasis via the induction of *SH3BP1* [141].

The role of the Hippo pathway in CRPC development and AR regulation recently gained momentum (Figure 4). Coimmunoprecipitation and immunofluorescence microscopy revealed that AR and YAP colocalize to and interact in the nucleus [98]. This interaction is androgen-dependent in LNCaP cells, but androgen-independent in C4-2 cells [98]. C4-2 cells are a hormone-independent subline of LNCaP cells, representing a clinical CRPC in vitro cell model [99]. Downregulation of YAP signaling results in the suppression of AR target genes, suggesting that YAP is critical for AR activity [98].

Interestingly, C4-2 cells harbor low MST1 kinase signaling, and restoring MST1 expression in these cells results in impeding the YAP–AR nuclear interaction and AR activity [98,100]. One plausible mechanism for AR upregulation in CRPC patients is, therefore, MST epigenetic silencing [101]. Cellular myelocytomatosis (c-MYC), a transcription factor that regulates cellular growth and proliferation [102], is commonly overexpressed in PCa patients, which induces tumor initiation [103]. Mechanistically, c-MYC is a regulator of the enhancer of zeste homolog 2 (EHZ2), which is a subunit of polycomb repressive complex 2 (PRC2) [104]. EZH2, a histone methyltransferase, catalyzes the trimethylation of histone 3 at lysine 27 (H3K27me3) to regulate gene expression [105]. EZH2 functions both as a transcriptional activator and repressor for specific gene sets in a cell-context-dependent manner [106]. EZH2 acts as a coactivator of the androgen receptor in CRPC [107]. c-MYC induces EZH2 activity via suppressing microRNA (miR)-26a/b, which results in MST1 promoter silencing [101]. Treating C4-2 cells with JQ1 results in downregulating c-MYC, which in turn induces MST1 expression and decreases cell survival [101]. However, combining MST1 knockdown with either c-MYC inhibition by 10058-F4 or EZH2 inhibition by GSK126 restores cell survival [101].

LATS2 and AR were, using immunohistochemistry, reported to colocalize within healthy prostate epithelium patient samples [108]. When in the nucleus, LATS2 and AR form a protein complex, which binds to *prostate-specific antigen (PSA)* promoter and enhancer regions [108]. LATS2 suppresses AR activity through hindering the NH_2_- and COOH-terminal interaction within the receptor [108]. The activation status of LATS2 was not examined, and whether AR is a direct substrate for LATS2 is unknown; it is, therefore, still an outstanding question if this LATS2-mediated regulation of AR transcription is phosphorylation-dependent [108]. Importantly, LATS2 levels negatively correlate with PCa tumor stage, a conserved phenomenon with several other types of carcinomas [108,109,110]. Paradoxically, *LATS2* is in a range of cell types and, in vivo, a YAP/TAZ–TEAD target gene [111,112,113], which forms an integral component of a feedback loop that keeps YAP/TAZ–TEAD activity levels in check [111,112,113]. Loss of LATS2 expression, but high YAP activity [75,98] and, therefore, impaired Hippo pathway feedback in high-grade PCa might, therefore, be a defining PCa hallmark. The relatively low LATS2 levels in PCa might be due to additional YAP/TAZ–TEAD-independent transcriptional regulation and/or post-transcriptional regulation of LATS2 protein. It will be critical to establish if negative feedback loops within the Hippo pathway are prevalent in healthy prostates and, if so, why these dynamic negative feedbacks might be defective in PCa. Therapeutically reinstating these negative feedback loops within the Hippo pathway might then be a viable option. Overall, these reports show that the Hippo kinase cascade and its effector YAP are regulators of AR nuclear localization and activity.

### 3.3. The Hippo Pathway, Tumor Microenvironment, and Immune Response Evasion

Cross-talk between the Hippo pathway and the tumor microenvironment is widespread across multiple solid tumors and regularly operates via a feed-forward loop that drives tumor progression [21,26,114,115]. YAP/TAZ is a signaling nexus and regulates cell–cell interaction and cell–stroma interaction through inducing the expression of a range of secretory proteins such as CYR61 and CTGF [31,54,115,116], as well as of components essential for mechanoresponsive plasma membrane organelles such as caveolae [117], and components and regulatory elements of focal adhesions such as integrins and cytoskeletal tension [54,118,119,120,121,122]. YAP/TAZ are well-established molecular sensors of the extracellular matrix (ECM), and both sense the stiffness and composition of the ECM [31,115,118]. In vitro experiments show that cells cultured on high ECM matrix stiffness result in increased YAP/TAZ nuclear localization and target gene expression [31,115,116,123,124]. In comparison, cells grown on low ECM stiffness have a higher cytoplasmic fraction of YAP/TAZ [31,115,116,123,124]. This is particularly important in PCa, as PCa is widely recognized for its rich tumor–stroma interaction [125,126].

Downregulation of α3 integrin causes PCa progression and promotes formation of metastatic lesions via altering YAP/TAZ activity. Mechanistically, loss of α3β1 in PCa results in the inhibition of the Abelson-related gene (Arg/abl2) tyrosine kinase cascade, which dephosphorylates the p190Rho-Guanosine triphosphate (GTP)ase activating protein-(GAP)/p120RAS-GAP (p190RhoGAP/p120RAS-GAP complex [127]. Consequently, Rho signaling is activated, ultimately causing increased YAP/TAZ levels, which promotes cellular migration in vitro and metastasis in vivo [127]. However, it is not entirely clear whether RhoA in this instance acts via the Hippo–LATS kinase cascade or independently from it. Paradoxically, α3β1 loss inhibited skin tumorigenesis in vivo [128]. Importantly, ECM regulates multiple cellular cancer properties [129] and it remains unclear whether these effects are mediated via the Hippo pathway in PCa and importantly whether ECM stiffness is inducing YAP activity in PCa. Addressing these questions might partly explain the increased YAP expression levels in PCa patients.

The ability of tumor cells to evade immune response is widely recognized to be a hallmark of cancer progression [130]. Intriguingly, YAP is partly responsible for this in PCa [131]. In a PTEN/ SMAD4 knockout PCa mouse model, YAP levels are elevated [131]. In this model, YAP expression results in myeloid-derived suppressor cell (MDSC) recruitment via the CXCL5/CXCR2 axis. MDSCs mediate tumor immune response evasion through suppressing T-cell activation, proliferation, and viability [132]. YAP–TEAD directly binds to the *CXCL5* promoter, inducing *CXCL5* expression. Either MDSC depletion, or inhibition of YAP or CXCL5/CXCR2 activity halts tumor progression [131]. Similarly, YAP hyperactivation is observed in the Kras/p53 knockout pancreatic cancer mouse model, which stimulates chemokines expression and thereby recruitment of MDSCs to tumors [133]. Of note, YAP governs the recruitment of tumor-infiltrating type II macrophages (M2) in liver carcinoma, which promotes tumorigenesis by avoiding immune clearance [134] (Figure 4). However, how the Hippo pathway gets dysregulated and drives PCa tumor–stroma interactions is still not fully understood.

### 3.4. TAZ’s Role in Metastasis

YAP and TAZ are modulators of cell motility and cytoskeletal dynamics in a feedback dependent manner [119]. TAZ in particular is a potent regulator of epithelial–mesenchymal transition (EMT) in most types of solid cancers, including ovarian cancer, glioma, and breast cancer [135,136,137,138,139,140]. The role of TAZ in PCa tumor progression and the regulatory nature between TAZ and AR is not well described. TAZ overexpression induces malignant transformation of the non-cancerous prostate epithelial cells, RWPE-1 [141]. Knockdown of TAZ in PCa cells causes reduction in migratory rate in two-dimensional (2D) cultures, as well as lower metastatic rate when injected in vivo. Endogenous expression of TAZ is regulated by ETS transcription factors members ETV1/4/5 (Figure 4) [141]. ETV1/4/5 induce TAZ gene expression, which results in the expression of SH3 domain-binding protein 1 (SH3BP1) via TAZ–TEAD. SH3BP1 belongs to the RhoGAP protein family and regulates Rac signaling to modulate cytoskeletal dynamics and cell motility [142]. So far, the PCa stage-specific levels of TAZ in PCa tumor samples are yet to be investigated.

## 4. The Hippo Pathway’s Role in Prostate Cancer Stem Cells

The development of CRPC following androgen deprivation therapy (ADT) is often inevitable [144]. CRPC likely develops from the prostate cancer stem cells (PCSCs), a subset of cells within the tumor which regulate initiation, but importantly also recurrence [145]. PCSCs were successfully isolated from patient tissue samples on the basis of their α_2_β_1_^hi^CD133^+^CD44^+^ phenotype [146,147,148,149]. PCSCs have a high proliferation rate and increased ability of colony formation in 3D cultures, as well as an ability to form prostate-like structures when injected in immunocompromised mice compared to CD44^−^ and CD133^−^ cells [146,147,148,149]. The Hippo pathway regulates cancer stem cells (CSCs) within a variety of tumors [136,150,151]. Interestingly, PC3 and DU145 cells resistant to the chemotherapeutic agent docetaxel possess a CD44^+^ phenotype. In this context, CD44 increases cellular migration rate in 2D cultures via inducing YAP, CYR61, and CTGF expression [152].

**Figure 5:**
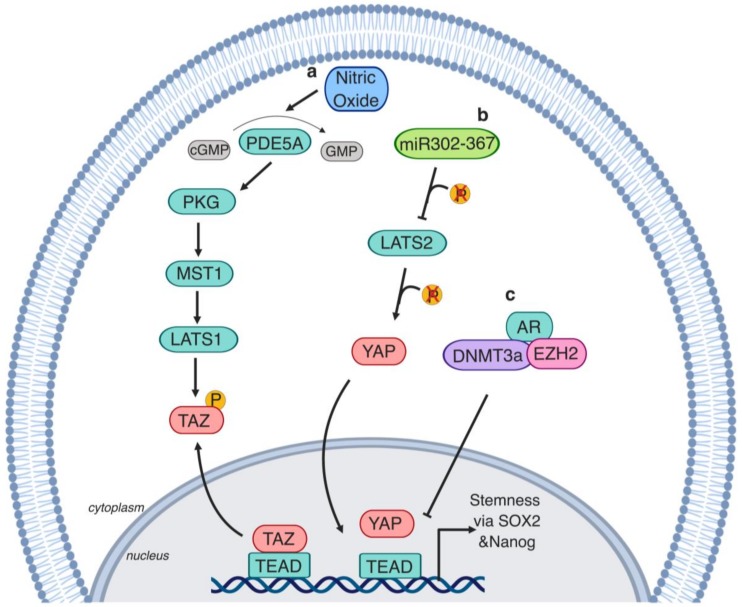
YAP/TAZ regulate prostate cancer stem cells (PCSCs). **a.** Inhibition of the stemness regulator cyclic GMP-specific phosphodiesterase type 5 (PDE5) by nitric oxide causes activation of cGMP-dependent protein G (PKG), which activates MST1/LATS1 and causes TAZ phosphorylation [154]. **b.** Stemness regulator microRNA (miR)-302–367 cluster induces LATS2 dephosphorylation which results in YAP nuclear translocation [153]. **c.** DNMT3a and EZH2 form a heterotrimeric complex with AR, which translocates to the *YAP* promoter inducing its silencing [155].

The stemness regulator microRNA, cluster miR-302–367, downregulates LATS2, which results in YAP dephosphorylation and nuclear translocation [153]. Additionally, miR-302–367 overexpression in LNCaP cells induces their capacity to form spheres in 2D cultures and xenograft tumors when injected into castrated mice [153]. Cyclic guanosine monophosphate (cGMP)-specific phosphodiesterase type 5 (PDE5) also induces stemness via the Hippo pathway [154]. Pharmacological PDE5 inhibition or inhibition via endogenous nitric oxide results in the activation of cGMP-dependent protein G (PKG); this activates MST1/LATS1 phosphorylation causing TAZ cytoplasmic retention and degradation [154]. AR further inhibits the transcriptional activity of YAP in LNCaP, as well as in the serially propagated castration-induced regression derived 22rv1 cells. Mechanistically, AR complexes with EZH2 and DNA methyltransferase 3 (DNMT3a) at the YAP promoter, causing its methylation and silencing [155]. In this sense, during androgen deprivation therapy, AR inhibition results in YAP transcriptional activation. YAP expression results in the transcription of stemness-stimulating genes in a TEAD-dependent manner, which induces sphere formation in vitro [155]. Additionally, inhibiting YAP activity in vivo prevents PCa recurrence in castrated TRAMP mice [155] (Figure 5).

## 5. Targeting the Hippo Pathway for Prostate Cancer Therapy

### 5.1. Targeting YAP/TAZ–TEAD

The Hippo pathway is a critical regulator of several hallmarks of PCa. Targeting Hippo–YAP/TAZ clinically, therefore, has therapeutic potential. As YAP/TAZ are transcriptional coactivators that principally function via binding to the TEAD family of transcription factors [52,53,156,157,158], the most direct route to target the Hippo pathway is via this interaction [158]. Verteporfin is a small-molecule inhibitor of this YAP/TAZ–TEAD interaction [159]. Verteporfin suppresses CRPC tumor growth and PCSC proliferation, which ultimately also prevents recurrence [75,98,155]. Although Verteporfin is used for macular degeneration treatment, its future use for cancer therapeutics is hampered by Verteporfin’s low solubility and low target affinity, which makes it generally toxic [160,161]. Vestigial-like 4 (VGLL4) is a tumor suppressor that competitively binds to TEAD via its tondu domain (TDU), thereby preventing YAP from mediating transcription [162,163]. VGLL4-mimicking peptide (super TDU) abrogates YAP binding to TEAD4, which has anti-tumor effects in gastric cancer patient-derived cells and in vivo in the gastric cancer mouse model driven by *Helicobacter pylori* infection [164]. A YAP-like peptide (17-mer) was designed aiming to impede YAP–TEAD binding. The 17-mer peptide has higher affinity for TEAD1 compared to YAP [165,166]. Although targeting the YAP–TEAD interaction appears to be the most straightforward route toward targeting the Hippo pathway, to date, none of the discovered agents are approved for cancer therapeutics.

### 5.2. Statins

Statins are a class of US food and drug administration (FDA)-approved drugs for hypercholesterolemia treatment [167]. Statins inhibit the enzyme, 3-hydroxy-3-methyl-glutaryl-coenzyme A (HMG-CoA) reductase, which prevents the conversion of HMG-CoA to mevalonic acid [168]. Subsequently, statins reduce the synthesis of geranylgeranyl pyrophosphate, which is required for Rho GTPase activity [169,170]. Statins induce YAP phosphorylation through Rho GTPase activity and actin rearrangement [169,170]. In vitro, statins induce gap 1 (G1) cell-cycle arrest and apoptosis in the PCa cell line, C4-2B [171]. Importantly, retrospective studies in a large Taiwanese cohort of statin-treated heart disease patients showed decreased incidence of PCa [172]. Furthermore, statins were recently identified to reduce PCa aggressiveness and metastasis incidence significantly in a retrospective study of a large cohort of Saskatchewan men [173]. Similarly, the occurrence of breast, ovarian, colorectal, and liver cancer is also reported to be lower in statin users [167]. Nonetheless, it remains unclear whether this statin-based clinical manifestation is mediated via the Hippo pathway.

### 5.3. Hippo Kinase Activators

The rapidly accelerated fibrosarcoma (RAF) family of serine/threonine kinases acts upstream of the MST kinases [174]. RAF-1 suppresses apoptosis by sequestering and preventing MST2 phosphorylation [174]. Inhibition of RAF-1, therefore, results in the activation of MST2. ISIS 1532 oligonucleotide was designed to target the 3′ untranslated region of cRaf messenger RNA (mRNA) [175,176]. In preclinical trials, ISIS 1532 inhibited lung carcinoma in in vivo mouse models [175,176]. However, three phase II clinical trials in patients with advanced PCa, and ovarian and colon cancers showed no significant response, and the agent was withdrawn from further testing [176,177,178,179]. Targeting the Hippo pathway kinases proves challenging as it is regulated by a variety of external cues and interacts with multiple signaling pathways [161]. In essence, activators of the YAP/TAZ inhibitory kinases are needed, and designing kinase activators is in general more challenging than inhibitors [180,181].

## 6. Signaling Cross-Talk between the Hippo Pathway and Multiple Signaling Pathways

### 6.1. WNT Receptor Signaling

Upon androgen deprivation, WNT signaling stimulation triggers the nuclear translocation of AR and YAP to the nucleus, which induces AR-mediated gene expression independently from β-catenin translocation [143] (Figure 4). YAP and TAZ are downstream effectors of WNT/β-catenin [182,183]. Upon WNT stimulation, YAP and TAZ are released from the WNT destruction complex and translocate to the nucleus to induce transcription [182,183]. Additionally, WNT-mediated activation of YAP/TAZ can occur independently from β-catenin via the scaffold protein, adenomatous polyposis coli (APC), which facilitates SAV1 and LATS1 phosphorylation via glycogen synthase kinase 3 β (GSK-3β) activity [184]. Importantly, APC activation mutations are reported in 5% of PCa patients [185]. Contradictorily, knockout of APC in vivo in mouse models results in prostate tumor formation [186]. Impressive work using an array of CripsR knockout cell lines, as well as mouse models, showed that alternative WNT signlling (Wnt5a/b) activates YAP/TAZ via GPCR α12/13; these G-coupled proteins signal to activate RhoGTPases that inhibit LATS1/2 activity [139,187]. Whether APC or alternative WNT signaling activates YAP/TAZ in PCa and whether this mechanism is mediated via androgen receptor signaling remains unexplored.

### 6.2. Mechanistic Target of Rapamycin (mTOR) Signaling

The mTOR protein is a central cell growth regulator, which is regulated by growth factors, energy levels, and nutrients. When active, mTOR stimulates biosynthetic pathways including nucleotide, protein, and lipid synthesis, while inhibiting catabolic processes, such as autophagy [188,189]. Intriguingly, PCa tumor cells with high PI3K/AKT/mTOR activity are proposed as a mechanism for prostate tumors to surpass hormone inhibition therapy [190,191,192]. Additionally, speckle-type POZ (pox virus and zinc finger protein) protein (SPOP) mutations, the most common mutations in primary PCa (10%) [74], induce PCa tumorigenesis via PI3K/mTOR [193]. Interestingly, YAP activates mTORC signaling in breast epithelial MCF10A cells. Mechanistically, YAP suppresses PTEN activity via miR-29 induction [194]. Consequently, in a transgene YAP mouse model, mTOR was activated, causing skin hyperplasia [194]. One of the strongest regulators of mTOR activity is amino-acid sensing; when amino-acid availability of specific amino acids is low, mTOR is switched off [189]. YAP/TAZ–TEAD induce the expression of a range of cellular amino-acid transporters [195,196], including the high-affinity hetero dimeric leucine transporter, LAT1 (encoded by *SLC7A5* and *SLC3A3*). Expression of LAT1 results in increased uptake of leucine at nutrient-limiting conditions [195], as is prevalent in tumors. Consequently, the expression of amino-acid transporters activates mTOR [195,196,197]. These mechanisms thereby provide a metabolic advantage for tumor cells with hyperactive YAP/TAZ. Furthermore, integrin α3 controls YAP phosphorylation and nuclear localization via the focal adhesion kinase/cell division control protein 42/protein phosphatase 1A (FAK/Cdc42/PP1A) axis, which activates mTOR [198]. Although these studies were not carried out in the context of PCa, they did indicate that YAP/TAZ–TEAD activity might be triggering the activation of mTOR in PCa. Remarkably, PTEN is a negative regulator of YAP activity in the PCa PTEN/SMAD4 knockout mouse model [131]. It is, therefore, a relevant outstanding question as to whether there is a feedback loop between PTEN suppression and YAP activity in PCa. Additionally, it remains unclear whether resistance to hormone inhibition therapy occurs via positive selection of cells with high YAP activity, which in turn induce tumorigenesis synergistically via TEAD binding and PI3K/mTOR activation.

### 6.3. Activator Protein (AP-1)

The activator protein (AP-1) transcription factor consists of dimeric complexes, which include the DNA-binding protein families, cellular ju-nana (c-Jun), cellular FBJ osteosarcoma oncogene (c-Fos), activating transcription factor (ATF), and cellular musculoaponeurotic fibrosarcoma (c-MAF) proteins [199,200]. AP-1 activation is mediated via a range of paracrine signaling molecules, as well as by the mitogen-activated extracellular signaling responsive kinase kinases (MEKs) [199,201,202]. AP-1 regulates multiple cellular responses such as inflammation, proliferation, and apoptosis [200,203]. Importantly, genome-wide analysis revealed that YAP/TAZ–TEAD mediate tumorigenesis by co-occupying the same genomic region occupied by AP-1 [204,205]. YAP/TAZ–TEAD and the AP-1 interaction occurs with the aid of the p160 family of steroid receptor co-activators (SRC1-3) [206]. Interestingly, SRC-3 is overexpressed in PCa, which promotes cell proliferation via AR activation [207]. Treating LNCaP cells with DHT induces the activity of c-Jun and tumor necrosis factor alpha (TNF-α) promoter activity, which contains AP-1 binding sites. In this context, the activation of AP-1 due to AR induction might synergistically be prompting YAP/TAZ target gene transcription [208]. These findings provide evidence for cross-talk between YAP/TAZ–TEAD and AP-1. Nonetheless, further investigation is required to understand the role of this signaling cross-talk in PCa development and its role in regulating AR.

## 7. Conclusions and Perspectives

To date, there are no identified somatic mutations of the Hippo pathway components in PCa. Furthermore, although YAP was identified to be amplified in a subset of PCa [209], it is evident that YAP/TAZ is a much more widespread contributor to PCa development. YAP and TAZ play key roles in multiple stages of PCa initiation, development, and progression, as well as regulation of AR signaling. However, the mechanistic insights into how YAP/TAZ becomes hyperactivated, how YAP/TAZ interacts with the stroma and their precise role in PCa development are currently far from fully elucidated. Obtaining further fundamental understanding of the complexity of YAP/TAZ hyperactivation in PCa onset and development is, therefore, crucial for improving future clinical interventions and care for PCa patients [210,211].

- How does YAP drive CRPC development? Androgen receptor bypass is a contributing mechanism via which PCa cells develop castration resistance [212]. Androgen-deprived PCa cells activate a variety of hormone receptors such as glucocorticoid receptor (GR) and its targets in order to overcome androgen dependence [212]. Importantly, GR signaling activates YAP in MDA-MB-231 breast cancer cells [213]. Additionally, the perplexing ability of tumors to activate steroidogenesis pathways causing AR hypersensitivity is not completely understood. Of note, YAP regulates steroidogenesis in ovarian granulosa cells [214]. Whether YAP is involved in inducing CRPC via AR bypass and intratumoral steroidogenesis, and whether YAP is essential for CRPC PCa cell survival are, to a great extent, still unexplored questions.
- The estrogen receptor (ER) plays an important role in PCa [215,216]. ERα regulates proinflammatory and pro-proliferative targets and is associated with high Gleason score [215,216]. In comparison, ERβ receptor plays an anti-inflammatory, pro-apoptotic role [215,216]. Estradiol, the estrogen receptor agonist, activates the Hippo pathway in the breast SK-BR-3 cell line via G-protein-coupled estrogen receptor (GPER) [217]. Although anatomically distinct, the molecular and clinical similarities between breast and prostate cancer [217] highlight the importance of examining if a similar cross-talk mechanism is occurring in PCa.
- Activation of the Hippo kinase cascade module is a clear direction toward utilizing the Hippo pathway therapeutically [161,181]. However, an ongoing challenge of this route is the complexity of the Hippo pathway upstream regulators. Intriguingly, in PCa, it is unclear what causes the Hippo pathway dysregulation. Delineating the upstream regulators of the Hippo pathway in a PCa-specific context might, therefore, have direct clinical relevance. Importantly, YAP is upregulated in CRPC; therefore, developing YAP activity inhibitors is an equally important therapeutic direction. Successfully controlling YAP and/or TAZ activity state therapeutically would be an immense step toward developing a personalized therapeutic strategy in CRPC.

## Abbreviations

ADT	Androgen deprivation therapy
AMOT	Angiomotin
AMPK	5′ adenosine monophosphate-activated protein kinase
AP-1	Activator protein 1
APC	Adenomatous polyposis coli
aPKC	Atypical protein kinase C
AR	Androgen receptor
Arg/abl2	Abelson-related gene
ATF	Activating transcription factor
CD133	Cluster of differentiation 133
CD44	Cluster of differentiation 44
Cdc42	Cell division control protein 42
c-Fos	FBJ osteosarcoma oncogene
cGMP	Cyclin guanosine monophosphate
c-Jun	Cellular ju-nana
c-MAF	Musculoaponeurotic fibrosarcoma
c-MYC	Cellular myelocytomatosis
CRPC	Castration-resistant prostate cancer
CTGF	Connective tissue growth factor
CXCL5	C–X–C motif chemokine 5
CXCR2	C–C chemokine receptor type 2
CYR61	Cysteine-rich angiogenic factor
DHT	Dihydrotestosterone
DNMT3a	DNA methyltransferase 3
ECM	Extracellular matrix
EMT	Epithelial–mesenchymal transition
ER	Estrogen receptor
ERG	ETS-regulated gene
ETS	E26 transformation-specific transcription factors
ETV1/4/5	E26 transformation-specific variant 1/4/5
EZH2	Enhancer of zeste homolog 2
FAK	Focal Adhesion Kinase
FDA	US food and drug administration
FRDM6	FERM domain-containing protein 6
GAP	Guanosine triphosphate (GTP)ase activating protein
GTP	Guanosine triphosphate
GR	Glucocorticoid receptor
HMG-CoA	3-hydroxy-3-methyl-glutaryl–coenzyme A
Hsp27	Heat shock protein 27
JMJD2A	Lysine-specific demethylase
JNK	c-Jun N-terminal kinase
KIBRA	Kidney- and brain-expressed protein
LATS1/2	Large tumor suppressor 1/2
M2	Tumor infiltration type II macrophages
MAP4K	MAP kinase kinase kinase kinases
MDSCs	Myeloid-derived suppressor cells
miR302-367	microRNA cluster 302-267
MOB1	MOB kinase activator 1
MST1/2	Mammalian Hippo homolog (Ste20-like kinases)
mTOR	Mammalian target of rapamycin
NF2/Merlin	Neurofibromatosis 2
NLK	Nemo-like kinase
Par3	Polarity protein 3
PCa	Prostate cancer
PCSCs	Prostate cancer stem cells
PDE5	Cyclic GMP-specific phosphodiesterase type 5
PI3K-AKT	Phosphoinositide 3-kinase/protein kinase B
PKG	cGMP-dependent protein G
POZ	Pox virus and zinc finger protein
PPA1	Protein phosphate 1
PRC2	Polycomb repressive complex 2
PSA	Prostate-specific antigen
PTEN	Phosphatase and tensin homolog
RAC	Ras-related C3 botulinum toxin substrate 1
RAF	Rapidly accelerated fibrosarcoma family of serine/threonine kinases
RhoGAP	Rho family of GTPases
SAV1	Protein salvador homolog 1
SH3BP1	SH3 domain-binding protein 1
Super TDU	VGLL4-mimicking peptide
TAZ	Transcriptional co-activator with PDZ-binding motif
TEAD1-4	TEA domain family member 1–4
VGLL4	Vestigial-like 4
WNT	Wingless
YAP	Yes-associated protein
17-mer	YAP-like peptide
